# Prioritising Housing Maintenance to Improve Health in Indigenous Communities in NSW over 20 years

**DOI:** 10.3390/ijerph17165946

**Published:** 2020-08-16

**Authors:** Jeffrey C. Standen, Geoffrey G. Morgan, Tim Sowerbutts, Katrina Blazek, Jessica Gugusheff, Otto Puntsag, Michael Wollan, Paul Torzillo

**Affiliations:** 1 Health Protection NSW, St Leonards NSW 2065, Australia; Otto.Puntsag@health.nsw.gov.au; 2 School of Public Health, Faculty of Medicine and Health, University of Sydney, Camperdown NSW 2006, Australia; geoffrey.morgan@sydney.edu.au (G.G.M.); Paul.torzillo@sydney.edu.au (P.T.); 3 University Centre for Rural Health, Faculty of Medicine and Health, University of Sydney, Lismore NSW 2480, Australia; 4 Q Social Research Consultants Pty Ltd., Broadway NSW 2007, Australia; sbutts@qsrc.com.au; 5 NSW Ministry of Health, St Leonards NSW 2065, Australia; katrina.blazek@sydney.edu.au (K.B.); Jessica.Gugusheff@health.nsw.gov.au (J.G.); Michael.Wollan@health.nsw.gov.au (M.W.)

**Keywords:** housing for health, health hardware, housing quality, Indigenous housing, Aboriginal housing, health, evaluation, housing standards, home health and safety, healthy living priorities

## Abstract

Many studies document the relationship between housing quality and health status. Poor housing in Aboriginal communities continues to be linked to the compromised health status of Aboriginal Australians. The New South Wales (NSW) Housing for Health (HfH) program has been assessing and repairing Aboriginal community housing across the state for 20 years using a standardised intervention methodology that aims to improve the health of Aboriginal people in NSW by improving their living environments. Items are tested and repairs are prioritised to maximise safety and health benefits and measured against 11 Critical Healthy Living Priorities (e.g., safety, facilities for washing people and clothes, removing waste and preparing food). Descriptive analysis of data collected pre- and post-intervention from 3670 houses was conducted to determine the effectiveness of the program. Analysis demonstrated statistically significant improvements in the ability of the houses to support safe and healthy living for all critical healthy living priorities post-interventions. Trend analysis demonstrated the magnitude of these improvements increased over 20 years. In 24 communities (*n* = 802 houses) where projects were repeated (5–17 years later), results indicate sustainability of improvements for 9 of 11 priorities. However, the overall condition of health-related hardware in Aboriginal community housing across NSW pre-intervention has not significantly changed during the program’s 20 years. Results suggest a systematic lack of routine maintenance and quality control continues to be the overwhelming cause for this lack of improvement pre-intervention. Our evaluation of the HfH program demonstrated that fidelity to a standardised housing testing and repair methodology to improve residents’ safety and health can have sustainable effects on housing infrastructure and associated health benefits, such as a 40% reduction in infectious disease hospital separations. Housing and health agencies should collaborate more closely on social housing programs and ensure programs are adequately resourced to address safety and health issues.

## 1. Introduction

The World Health Organization (WHO) recognises poor housing as one of the main social causes of ill health [1,2] and extensive evidence has demonstrated improvements in health associated with improvements in housing and living environments since the late 1800s [3,4,5].

While Australia is an economically developed country with a high standard of living [6,7,8], poor housing in Aboriginal communities continues to be linked to the compromised health status of Aboriginal Australians since early last century [9,10,11,12,13,14,15,16,17,18].

There are around 800,000 Aboriginal and Torres Strait Islander people in Australia, representing 3.3% of the total Australian population. Whilst in remote areas there are higher proportions of Aboriginal Australians, one-third of the Australian Aboriginal population lives in New South Wales (NSW), the most populous state in Australia [19,20]. The NSW state health authority (NSW Health) has been delivering Housing for Health (HfH) projects with Aboriginal communities across NSW since 1997. HfH aims to improve the health status of Aboriginal people, particularly children, by assessing, repairing or replacing “health hardware” (particularly plumbing and electrical items) in houses to ensure they are safe and support occupants to practice healthy living. Health hardware in the context of HfH is defined as “the physical equipment needed to give people access to the health giving services of housing” [21].

HfH is a structured process for surveying and fixing houses, developed by Healthabitat, a not-for profit organisation, in the early 1990s [22,23]. It has since been used throughout Australia and internationally [9,24] and adopts a “no survey without service” approach to testing, recording, repairing and reporting at each survey. The “no survey without service” approach means no survey data is collected without a service being provided, such as making immediate repairs to items that require urgent attention [22].

In 1997 an interagency environmental health committee of NSW government agencies funded a trial HfH project in one discrete Aboriginal community in Northern NSW that demonstrated measurable improvement in the condition of those houses and ability to support healthy living. Consequently, a jointly funded program by NSW Health and the NSW Governments’ 10-year Aboriginal Communities Development Program (ACDP) expanded HfH to other NSW Aboriginal communities—usually a small town or a neighbourhood in a larger town [25]. An evaluation at the end of the ACDP funding cycle demonstrated positive health outcomes and subsequently the program was recurrently funded by NSW Health and funding increased. The NSW HfH program is managed centrally by NSW Health’s Aboriginal Environmental Health Unit and delivered jointly with regional NSW Public Health Units (PHUs), in partnership with the Aboriginal communities. The HfH program and financial management is guided by NSW state government policies and procedures.

This paper aims to describe the development of the HfH program in NSW, provide an overview of the program methodology and an analysis of program data over 20 years, and discuss the benefits and limitations of the program.

## 2. Methods

### 2.1. Data Collection

Housing for Health (HfH) projects (also referred to as the intervention) are run according to a standardised seven stage process [26,27] which involves the collection of data on house condition, remediation works completed and associated expenditure.

#### 2.1.1. Stages 1-3: Community Selection and Project Establishment

In NSW, projects are delivered continuously, and at any given time there are around 10–15 projects at various stages of implementation across the state. The selection of Aboriginal communities invited to participate in HfH (Stage 1) is primarily based on need determined from information on housing condition provided by PHUs, communities and government reports—and the availability of project resources at the time. Communities are not randomly selected.

Community consultation and a feasibility assessment (Stage 2) is undertaken to consider logistical issues in delivering the project—such as employment of local workers, insurances and accommodation—and to obtain community consent prior to proceeding. Consultation is undertaken with the housing providers, community leaders and each household. Project establishment (Stage 3) informs residents, arranges local work opportunities by employing local community workers and tradespeople, purchases consumables, and prepares for the immediate fix during the next stages.

#### 2.1.2. Stage 4: Baseline Survey and Urgent Repairs

Teams of trained community workers led by qualified team leaders, usually from local PHUs, undertake the initial baseline survey and fix (SF1). SF1 tests and records 268 items in each house using standardised, repeatable tests based on Australian standards and current best practice. The raw survey items provide useful information on specific issues in houses such as number of residents, number of taps working and hot water temperatures, and guides the repair works. Teams carry out minor repairs immediately such as replacing light globes and shower heads or unblocking sinks. The tradespeople—licenced plumbers and electricians—follow the teams within the next few hours repairing urgent and more complex items identified by the survey such as replacing hot water systems. There are some widely held beliefs about social housing being poorly looked after by the tenants, so the tradespeople are required to identify and record the cause for each repair being either:

- *Routine* (maintenance reasonably expected in a house);
- *Faulty* (the item isn’t present or installed incorrectly), or
- *Damage* (by people, and not by ants, vermin, poor water quality or other factors outside of the residents’ control).

#### 2.1.3. Stage 5: Major Repairs

Larger, more complex works such as re-waterproofing showers or improving accessibility for elderly or disabled tenants are implemented over the next 4 to 18 months following SF1, with the length determined by the number and condition of houses.

#### 2.1.4. Stages 6 and 7: Follow-Up Survey and Final Report

Following the completion of the major repairs, a second survey and fix (SF2) is undertaken using the same survey instrument as SF1. This second survey and fix provides a data driven comparison between SF1 conducted *before* any works completed and SF2 conducted *after* all works are completed. This is usually between six and 18 months later, depending on the amount of work required. SF2 facilitates repairs to any outstanding items since SF1 and enables an audit of the works in Stage 4 and Stage 5 by community teams. A report of the housing condition before and after each project, and a list of all works undertaken—by house and by trade—is compiled for each HfH project and provided to the community organisations at the completion of the project.

#### 2.1.5. Repeat HfH Projects

As the program has been continuously delivered around the state of NSW for two decades, most of the larger Aboriginal community housing providers in both discrete and non-discrete Aboriginal communities have received a HfH project. In the second decade, some communities received a repeat HfH project. The time between first and second HfH projects ranges from five to 17 years, and timing was dependent on specific issues in those communities.

#### 2.1.6. Methods of Prioritising Repairs

The program has a limited budget, so all works are prioritised to maximise the safety and health benefit to householders. Immediate life-threatening dangers are addressed as the highest priority, followed by repairs that ensure the house structure and hardware can support healthy living by the residents. Based on the literature, Healthabitat developed a practical implementation of evidence-based practices that support healthy living [11,22]. These included 11 Critical Healthy Living Priorities (CHLPs) which are reported in this analysis:

1. Power, water, and waste connected
2. Electrical safety
3. Gas safety
4. Structure and access
5. Fire safety
6. Shower working adequately
7. Facilities to wash children
8. Laundry services
9. Flush toilet working
10. All drains working
11. Facilities to store, prepare and cook food

Work is allocated in accordance with these priorities, but not all may be met at the completion of a HfH project given funding limitations.

### 2.2. Statistical Analysis

There are 268 raw items collected at survey and 41 items calculated based on the survey items, giving a total of 309 items per house. These items have response codes assigned to them with a score for each response: typically, 1 for a pass and 0 for a fail, although some items have slightly more complex scores.

**Figure A2:**
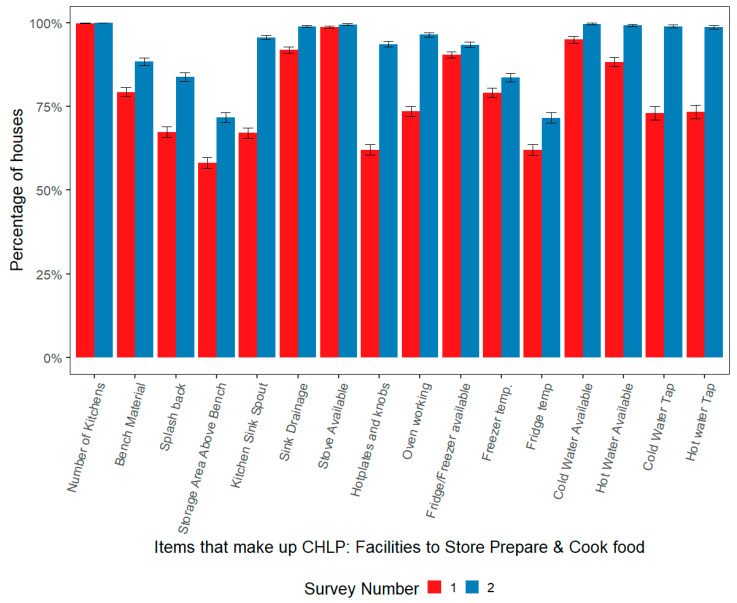
Percentage of houses where Critical Healthy Living Priority: *Improving Nutrition: Ability to Store, Prepare and Cook Food* was fully met for all NSW projects from 1998–2017.

Each CHLP is measured by assigning a set of data items from the survey. For example, for a house to enable residents to wash effectively and to maintain hygiene and health, a shower in a house requires the following minimum seven items to be functioning: hot water; cold water; hot water at a safe but effective temperature; hot and cold water taps; a shower hose and adequate drainage [9]. The CHLP score is calculated as the sum of scores for each item, with the maximum score being the sum of maximum possible scores for those items. (Figure A2 in Appendix B shows similar criteria to store, prepare and cook food.)

Two methods were used to calculate summary statistics for a set of houses:

1. The percentage of houses where CHLPs were fully met, i.e., a house’s score for a CHLP equaled the maximum possible CHLP score, therefore fully meeting the standard.
2. The percentage of houses where CHLP scores were within specified ranges i.e., Good (100% of maximum score); Fair (50% to 99% score) and Poor (less than 50%). This method was used for analysis in Appendix C.

Survey data from 112 HfH projects from 1998–2017 were analysed using SAS Enterprise Guide software, Version 9.4 [28]. Descriptive analyses were conducted of these CHLP summary measures collected at SF1 and SF2 to compare before and after house function within all 112 HfH projects and assess changes over 20 years.

The percentage of houses where a CHLP was fully met was calculated as a binomial proportion for all HfH projects in the relevant time period. A 95% confidence level interval for the binomial proportion was constructed using the normal approximation. To compare the percentages of CHLPs fully met between SF1 and SF2, the proportions of houses before and after intervention were tested for marginal homogeneity for matched pairs using the McNemar test statistic at 95% confidence level [29]. A matched-pairs method compares categorical responses for two samples when each observation (i.e., the house that received HfH) in one sample pairs with an observation in the other sample.

The HfH projects were grouped into 5-year intervals based on completion dates for each project and an average across the CHLP scores was calculated to determine trends over time. For each CHLP we assessed whether there was a linear trend in the percentage fully met across an ordered factor (5-year intervals). This analysis was applied separately at SF1 and SF2 and the Cochran–Armitage trend test statistic was used at 95% confidence level [29].

Separate analysis was undertaken for those communities that had a repeat HfH project (*n* = 802 houses, *n* = 24 projects) to compare CHLP scores over time within the same community.

## 3. Results

Over the 20-year period (1998–2017), the NSW Housing for Health (HfH) program conducted 113 HfH community projects (or interventions) in 3670 houses with a resident population of 14,609 people. In total, 88 communities (2791 houses) received at least one HfH project from 1998 to 2017. Of these 88 communities, 24 (802 houses) had a second project between five and 17 years later. One community had a third project implemented but it is not included in this analysis.

**Figure 1:**
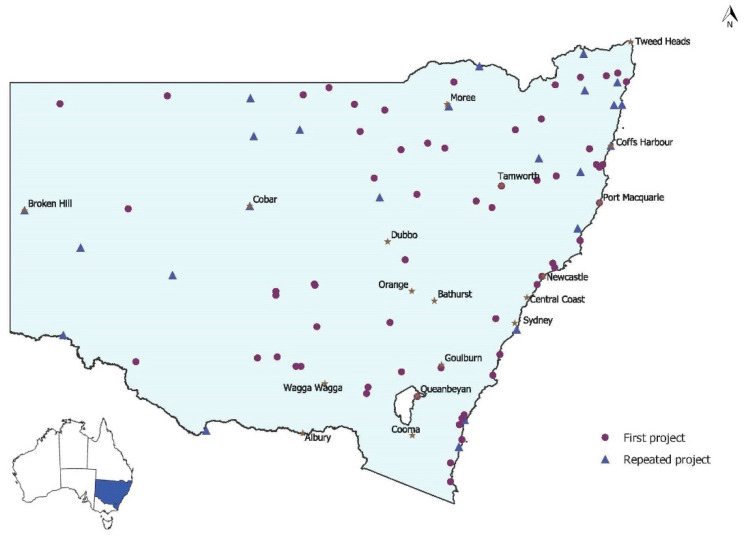
Location of New South Wales (NSW) Housing for Health Projects^+^ from 1998 to 2017. Note: ^+^ where two projects are in close proximity (e.g., in the same town), they may be represented by one triangle or circle.

Figure 1 shows the distribution of communities where HfH projects were conducted across NSW from 1998 to 2017, including those communities visited more than once. There is a wide distribution of HfH projects in communities across the state in urban, rural and remote areas. The HfH program has reached around 70% of houses in the NSW Aboriginal community housing sector over this time.

Table 1 summarises this information by five-year intervals. The results show the number of projects, houses and residents (recorded at SF1) in communities that received one or more HfH projects.

**Figure 2:**
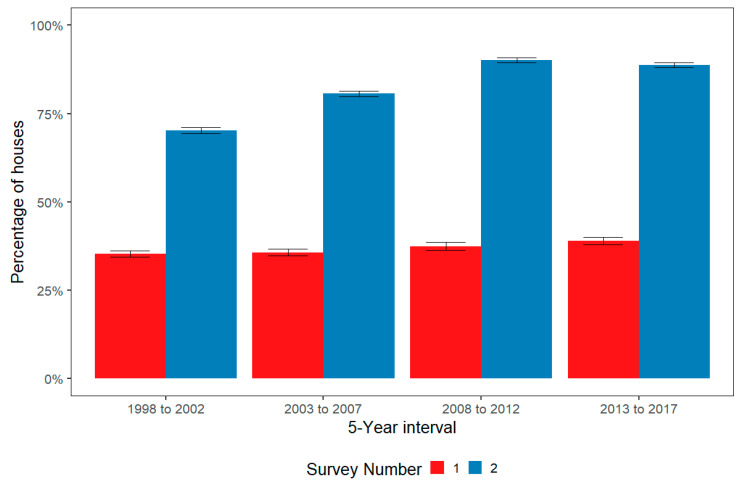
Average Percentage of houses with Critical Healthy Living Priorities fully met at Survey-Fix 1 and Survey–Fix 2 for all NSW projects 1998–2017 by 5-year intervals.

**Figure 3:**
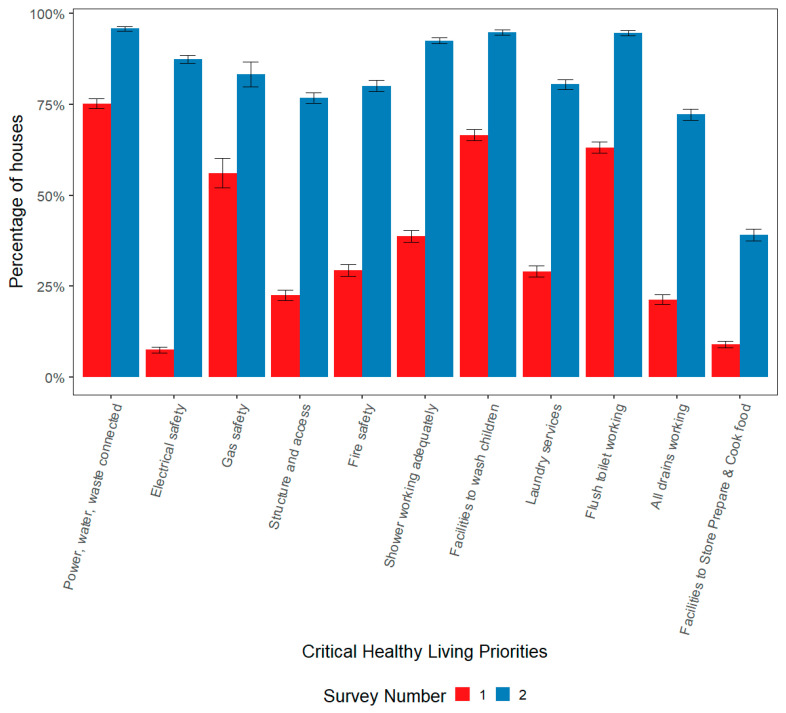
Percentage of houses with Critical Healthy Living Priorities fully met at Survey-Fix 1 and Survey–Fix 2 for all NSW projects (*n* = 112) from 1998–2017.

Figure 2 shows the average percentage of houses where all CHLPs were fully met at SF1 and SF2 for all NSW projects by 5-year intervals. (Table A6 in Appendix E summarises data for Figure 3). Trend analysis shows increasing improvement at SF2 over time. However, the CHLP related condition of houses before the project (SF1) remained consistently low (below 40% average across CHLPs) over the past 20 years. This general trend is reflected in nearly all the critical HLPs (represented in Appendix D).

Figure 3 shows changes in the CHLPs (the most important items needed to support safe and healthy living) between SF1 (red bars) and SF2 (blue bars) for all projects completed in NSW over the past 20 years. The CHLP categories are prioritised from left (highest) to right. (See Appendix E Table A5 for data tables for Figure 3). The columns represent the percentage of houses that met all criteria (100% score) for each CHLP category in all HfH projects (*n* = 112). If one or more criteria failed in a house, the house was not considered to adequately support that CHLP item.

At the initial survey (SF1), safety in houses was low, particularly electrical safety (7.5%), structure and access (23%) and fire safety (29%). For those houses with gas, just over half (56%) met the safety requirements. With regard to washing people, only 39% of all houses had all items in the shower working and two thirds of houses had a place to wash a small child with all hardware working (such as a bath, large basin or laundry tub with washing machine by-pass). Only 29% of houses had laundry facilities to support the washing of clothes and bedding. This includes the hardware and space to install a washing machine safely but does not include the washing machine itself as this is considered a tenant responsibility in social housing. Only two thirds of houses had flush toilets working properly, and 21% had all drainage working. Improving nutrition is assessed by whether houses support the ability of residents to prepare, cook and store food safely. Only 9% of houses had all items in this CHLP category working at SF1.

Chi-squared analysis (*P* < 0.01) revealed significant improvement in the percentage of houses meeting each CHLP from SF1 to SF2 in all categories. Following SF2, a score of at least 75% was attained for all but two of the CHLPs, with four CHLPs exceeding a score of 90%. The biggest improvement was in electrical safety, with only 7% meeting this CHLP at SF1 and 87% at SF2. Whilst the ability to store, prepare, and cook food was improved more than 4-fold, only 39% of houses had all items working at the end of the projects.

**Figure 4:**
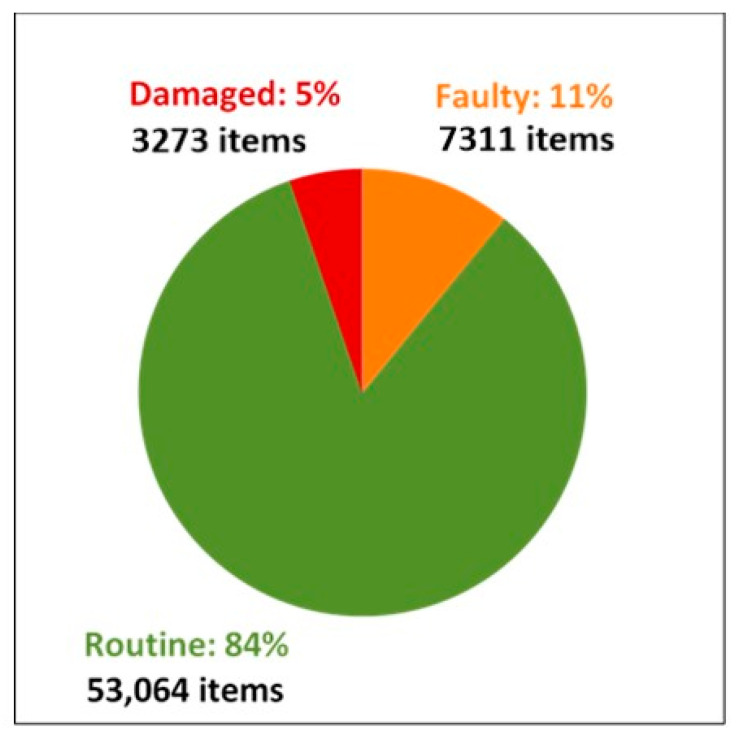
Percentage of items fixed under the NSW Housing for Health Program (*n* = 63,648) by the reason for repair from 1998–2017.

Figure 4 presents data on the reasons tradespeople recorded for repairing 63,648 items identified by the survey during the 20-year study period. Across NSW Aboriginal community housing, 84% of items repaired were routine maintenance issues. Faulty design or workmanship accounted for 11% of failures, with 5% of items fixed as a result of damage by the tenants.

**Figure 5:**
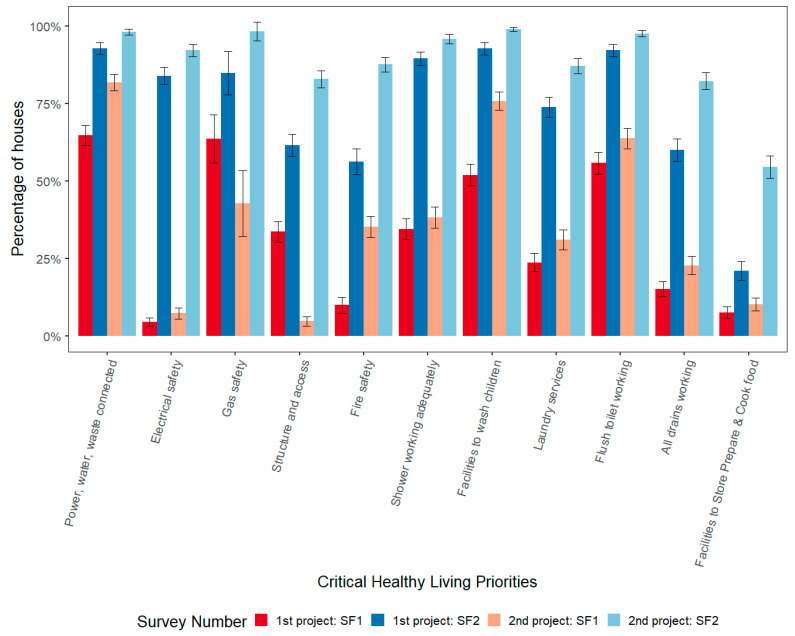
Percentage of houses with Critical Healthy Living Priorities fully met for NSW projects with repeat visits at First Project SF1 (*n* = 802) and SF2 (*n* = 722); and at Second Project SF1 (*n* = 788) and SF2 (*n* = 734) from 1998–2017.

Figure 5 shows the CHLPs for 24 communities where a repeat project was implemented in the second decade of the program. The red and dark blue bars show the percentage of houses meeting each CHLP at the first project SF1 and SF2, respectively. The orange and light blue bars show the percentage of houses meeting each CHLP at the second project SF1 and SF2, respectively. (See Table A7 in Appendix E for Data Tables for Figure 5).

In both visits, significant improvements in houses were made between SF1 and SF2. The SF2 results for all CHLPs in the second HfH projects within the same community were higher than SF2 in the first HfH projects in those communities. All CHLPs except *Gas Safety* and *Structure and Access* followed a similar pattern: the lowest house function was recorded at SF1 of the first HfH project. These increased significantly by SF2. In the five to 17 years between the first and second HfH projects in these communities, the functionality of the houses dropped, but overall not to the level of the first HfH project in these communities, indicating some of the improvements from the first project may have been sustained in these 24 communities that had a second HfH project.

## 4. Discussion

This report highlights the sustained improvements to housing within NSW Aboriginal communities as a result of the NSW Housing for Health (HfH) program over a 20-year period from 1998 to 2017. The research demonstrated significant improvements in the condition of houses and the ability to meet critical healthy living priorities (CHLPs) due to the HfH project intervention (Figure 3). Not all houses reached 100% for all the CHLPs reported in this paper, but more detailed analysis of the data showed there were still measurable improvements in each house (see Appendix C).

Most CHLPs achieved greater than 75% compliance after the HfH project intervention. However, results are generally lower for the lower priority CHLPs largely because of HfH program budgetary constraints restricting improvement on expensive items.

Each CHLP comprises a set number of items and a house must pass all these items to achieve that CHLP goal. Occasionally, achieving a maximum score for all CHLP items is beyond the scope of the HfH program funding and not all items are fixed. For example, fire safety in all houses was upgraded to current standards for smoke detection, but in some houses, security screens had been permanently fixed to the building frame, increasing security but preventing egress (fire escape) from windows.

Whilst the facilities to store, prepare and cook food were improved more than four-fold due to the HfH project intervention, only 39% of houses had all items working after the intervention. Budgetary constraints limited the ability to improve all items within this CHLP, particularly in the first decade of the program. Appendix B shows the separate criteria for this CHLP in more detail. Increased HfH funding allocation in the second decade of the program is likely to have contributed to the measurable improvement in this category over time (as shown in Appendix D).

The results of Survey-Fix2 (SF2) are a measure of the effectiveness of the HfH project intervention to improve house function over the past 20 years. Trend analysis of the overall SF2 data post-intervention (Figure 2) indicates the HfH program has become more effective over time. This is most likely a result of improved targeting of items for repair by HfH project managers and some increased funding. Appendix E shows that this same trend of program efficiency is consistent across each of the CHLPs.

Evaluation of house function is built into the methodology of the NSW HfH program for each HfH project but quantifying the impact of the HfH program on health outcomes is challenging. A 10-year evaluation of the program assessed changes in hospital admissions before and after each project. The analysis linked hospital admissions data to all houses in the HfH program over the first decade and demonstrated a 40% reduction in hospital separations for environmentally related infectious diseases for those residents of houses included in the HfH program compared to a control population [30]. A summary of this evaluation and these results is presented in Appendix A. Whilst this analysis cannot demonstrate causality between the HfH intervention and reduced disease, it does demonstrate a strong association between these improved house function measures and improved health outcomes [30]. The significant reductions in hospitalisations found in this assessment occurred in the context of significant improvement in targeted safety and healthy living practice measures over the first decade of the HfH program. Figure 2 demonstrates even greater improvements in house function over the second decade of the HfH program (2008 to 2017) compared to the first decade (1998 to 2007), indicating the same or possibly better health outcomes would likely be continued over the life of the HfH program.

Survey-Fix 1 (SF1) data describes of the condition of housing before any work was undertaken as part of the HfH intervention. The HfH program has surveyed and fixed around 70% of the NSW Aboriginal community housing sector, presenting a picture of the housing condition in the sector over 20 years. The ability of Aboriginal community housing in NSW to support basic safety and healthy living priorities prior to a HfH project was below 40% across all houses (Figure 2) and trend analysis indicates a lack of significant improvement in the condition of houses in the sector over the last two decades.

The failure of housing management systems is a likely reason for this lack of improvement in house function in Aboriginal communities over the past two decades (see Figure 4). NSW HfH program data indicates 95% of the repairs made on these houses resulted from a failure of systems to ensure routine maintenance (84%) and adequate checks on the quality of workmanship (11%). Focusing on tenancy management to reduce tenant damage will only address 5% of the issues related to house function in NSW community housing. Our results are consistent with previously published national data (2006) which showed, whilst there were slightly higher rates of tenant damage (10%), the primary cause for house function failure stemmed from a failure of maintenance regimes and quality control [9].

Although the average condition of houses at SF1 across all 112 HfH projects shows very little change over 20 years, (Figure 2) the 24 locations that have received a second HfH project have maintained higher house function (at the second project SF1) for most CHLPs (Figure 5), suggesting a sustainable benefit of the HfH program over time. This finding is consistent with results reported in the 10-year review which showed one community in 2003 where a third survey and fix had been undertaken 2–3 years later to gauge the sustainability of the program, house function had deteriorated slightly since SF2, but only 5% of the original funding was required to bring the houses back to the same standard [30]. Anecdotally, HfH project managers have reported that higher quality health hardware (e.g., taps) specified by the program was still functioning at the return visit. Whilst high quality materials may cost a little extra, they are likely an investment in sustainable health hardware. Further analysis of the survey and financial data for these 24 projects is planned to identify the sustainability of improvements in individual items.

Data from communities that received a repeat HfH project demonstrates significant improvement in the condition of the houses after each intervention (from SF1 to SF2). The improvement between SF2 results at the end of the first and second interventions is consistent with the general improvements in project delivery over the life of the program, illustrated in Figure 2.

The results of the HfH program assessment of evidence-based housing safety and health priorities presented here demonstrates significant improvements in the home environments over two decades. For disadvantaged families where unemployment is high, the home environment is often the environment where people spend most of their time. Ensuring the homes’ ability to support health is associated with significant reduction in the rates of infectious diseases, which in turn can reduce the risk factors for many chronic diseases, such as renal and cardiac disease, both of which are overrepresented in the Aboriginal population [17,20,31]. The HfH program also helps ensure the home environment supports practices delivered by health messages through clinical and population health services.

Improving health outcomes should reduce health expenditure on preventable conditions. “The cost of poor housing is borne by the health system” [32] and, while the extent of the financial benefit to health from the NSW HfH program is yet to be quantified, the relatively small amounts of HfH program funding that supports healthy living is likely to be an investment in health into the future. The benefits of the HfH program are not limited to improvements in house function and health outcomes. The strong engagement with Aboriginal communities throughout the process builds relationships between the communities and the NSW state health authority, as well as local Public Health Units. On the strength of these relationships, other issues of concern to the community, such as drinking water quality or waste management, have been raised and addressed by separate programs. Socioeconomic disadvantage covers a wide range of factors of which a functioning house may only be one. However, for a householder juggling many issues in the home, it can mean one less cause of stress and disempowerment in their life, allowing them the energy to focus on other issues including their health and the health of their family.

Much has been published on the connection of housing and health in the international literature. In the context of industrialised economies, much of the modern literature relates to urbanisation, energy efficiency, temperature control (particularly in cold environments), and indoor air quality issues such as mould and chemical exposures [33,34,35,36]. There is also a considerable body of literature on improving drinking water, sanitation and hygiene (WaSH) in the developing country context [37,38,39,40,41,42]. There is less published internationally on the capability of modern housing to support issues such as safety and WaSH principles. In the Australian context, a number of housing-related papers in the published and grey literature reference safety and healthy living practices as a measure or a best practice standard for housing [17,43,44,45,46,47,48], but the findings of this paper suggest no assumptions can be made that are adequately addressed in modern public housing, especially in Indigenous communities.

## 5. Strengths and Limitations

A strength of the HfH program has been the consistent collection of detailed data on houses in Aboriginal communities over an extended period. Whilst the primary purpose of the data is to guide repair work for each HfH project, the consolidated data provides evidence that can inform social housing policy and program delivery. Despite the gains demonstrated over the past 20 years, the HfH program does not address all issues in the homes. An average budget of only $9220 per house (Consumer Price Index (CPI) adjusted) has limited the HfH program’s ability to improve all items in houses, and more detailed analysis of the HfH program data may help identify specific items for repair that provide further improvements to the CHLPs.

The HfH program does not focus on social housing issues such as tenancy and long-term asset management. Its primary focus is on improving health by providing a level of attention to detail on hardware items that improve safety and health in houses, and these items are a basic standard that could be applied to all social housing. Telfar-Barnard et al. described a household “warrant of fitness” assessment for all rental housing in New Zealand to ensure basic measures of habitability are met, [49] similar to a basic standard of testing applied to car registrations in many jurisdictions. A similar approach could be applied to social housing in Australia to include criteria that ensure houses support basic healthy living practices in any handover or annual inspection processes.

## 6. Conclusions

Analysis of the Housing for Health (HfH) program data has demonstrated significant improvements in critical healthy living priorities in Aboriginal community housing in NSW where the program has been implemented. The magnitude of these improvements has increased over 20 years and results indicate sustainability of improvements for most priorities. The NSW HfH program’s collection of consistent data over this time provides a repository of information which can guide future policy and program development. Despite the benefits from the HfH program, across NSW there has been little change over two decades in the poor standard of safety and health hardware in Aboriginal homes prior to the HfH projects. A lack of routine maintenance and faulty design or construction is overwhelmingly the key cause identified for the failure of items repaired under the program. This data suggests the systems for maintenance of health hardware in Aboriginal community housing over the past 20 years have not improved healthy living priorities. This lack of improvement may be contributing to the gap in Aboriginal health compared to the rest of the Australian population. The specification of quality health hardware in maintenance programs is likely to be a cost-effective investment in both housing and health in the long term. Fidelity to a detailed HfH program methodology of standardised testing and repair of defined items that improve safety and health has led to these significant and sustainable improvements in house function in Aboriginal communities over the past 20 years of the HfH program in NSW. The effectiveness of the HfH program has improved significantly over the second decade, suggesting the substantial improvements in health outcomes associated with the first decade of the HfH program [30] will likely be sustained or increased. The program should be expanded to communities that have not yet received the program, and the program principles should be embedded into larger social housing repair and maintenance programs.

## Figures and Tables

**Table 1:** Summary results of Housing for Health program data by 5-year intervals.

Period		Project	Houses	Residents *
1998–2002	Visit 1	35	1141	5021
	Visit 2	0	0	0
	Total	35	1141	5021
2003–2007	Visit 1	28	911	3621
	Visit 2	1	16	51
	Total	29	927	3672
2008–2012	Visit 1	11	356	1335
	Visit 2	8	327	1339
	Total	19	683	2674
2013-2017	Visit 1	14	383	1385
	Visit 2	15	459	1507
	Total	29	842	2892
All years	Visit 1	88	2791	11,362
	Visit 2	24	802	2897
	Total	112	3593	14,259

* The number of people living in a house is recorded during the Survey Fix 1.

**Figure A1:**
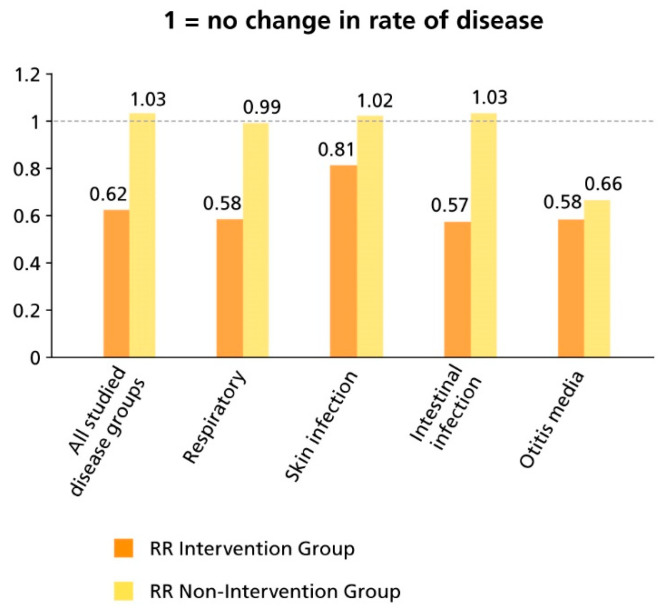
Before and after rate ratios for disease conditions in HfH intervention and non-intervention groups during a Health Outcomes Evaluation of Housing for Health.Source: Aboriginal Environmental Health Unit, Closing the Gap: 10 Years of Housing for Health in NSW—An evaluation of a healthy housing intervention. 2010: Sydney, NSW.

**Figure A3:**
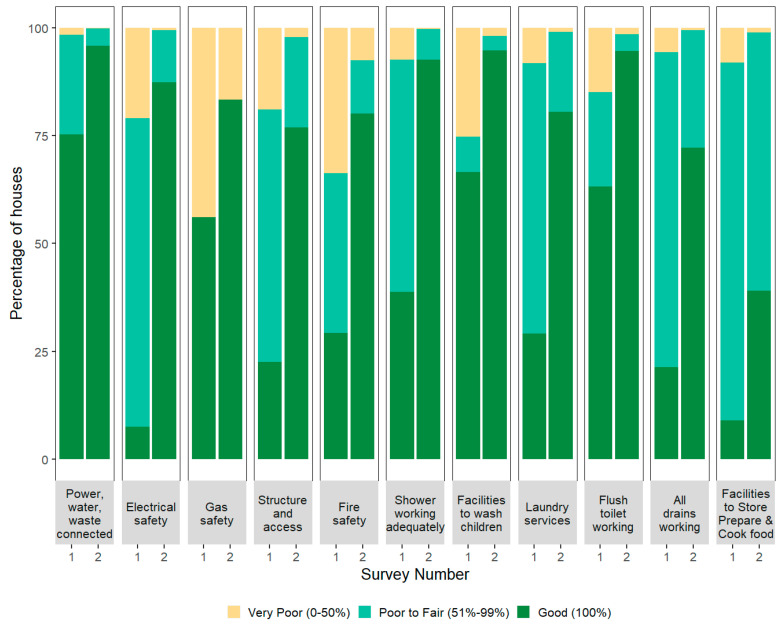
Comparison of Survey-Fix 1 and Survey-Fix 2 data for each critical Healthy Living Priority by category of house condition (Good, Fair or Poor) based on house function from 1998–2017.

**Figure A4:**
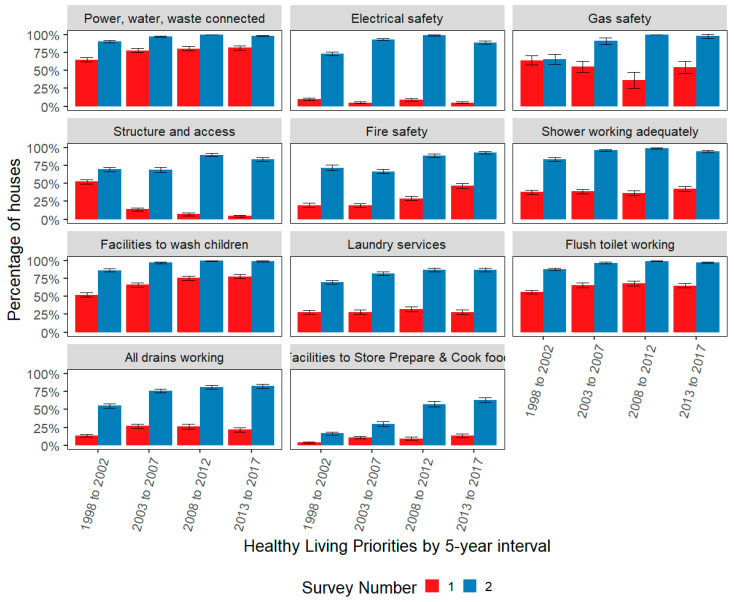
Percentage of houses where Critical Healthy Living Priorities were fully met at Survey-Fix1 and Survey–Fix 2 by 5-year intervals from 1998–2017.

## Appendix A. Health Outcomes Evaluation of the NSW Housing for Health Program

A health linkage evaluation of the New South Wales (NSW) Housing for Health (HfH) program was conducted after the first 10 years of the program. The evaluation looked at the impact of the HfH program on World Health Organization’s (WHO) International Statistical Classification of Diseases and Related Health Problems (ICD) Revision 10 (ICD-10) disease classifications attributed to changes to the home environment.

**Table A1:** List of International Statistical Classification of Diseases and Related Health Problems Revision 10 (ICD-10) codes for diseases related to the home environment (used for evaluation of health outcomes from Housing for Health).

Diseases	ICD-10 Codes
Acute respiratory Infections	J00-J22
Hepatitis—Viral	B15-B19 O98.4, P35.3
Intestinal Infectious Diseases	A01-A09
Otitis media	H65.1, H65.2, H65.3, H66, H67.0, H67.8
Skin infections	L00-L08

The evaluation linked the location of houses that received a HfH project between 1998 and 2008 to usual residential addresses data in the NSW Health routinely collected hospital admissions data. The rate of disease-specific hospital admission was calculated for each month in each community over the 10-year study period. Descriptive analysis assessed the rate of hospitalisations before and after each HfH project by calculating disease-specific rate ratios for each project and collectively for the whole program. The same process was used to calculate the disease-specific rate ratios for the control population (defined as the NSW rural Aboriginal population that did not receive the HfH intervention). The ratio of the two rate ratios was then calculated to assess any impact the program had on the selected hospitalisations over the 10 years [30]. Figure A1 shows these rate ratios for both case and control groups by grouped categories: All studied disease groups; Respiratory; Skin; Intestinal infections (including hepatitis) and Otitis media [30].

Routinely collected hospital separations data were used in the analysis due to its availability; however, it should be noted that for many home environment-related infections, hospital admissions would only represent the most serious of cases. Some symptoms may not be treated, and most infectious eye, ear, skin, respiratory or gastro-intestinal conditions would likely be treated at a primary health service such as a medical service or local general practitioner. As only the most serious cases would require hospital admission, these data may only represent a small portion of the overall burden of environmentally related diseases associated with Aboriginal community housing.

## Appendix B. Criteria for the Critical Healthy Living Priority, *Improving Nutrition: Ability to Store, Prepare and Cook Food*

Each of the critical Healthy Living Priorities (CHLP) presented in the Housing for Health program results are measured by assigning a set of data items for that particular CHLP. Figure A2 shows the separate items for the CHLP: *Improving Nutrition: Ability to Store, Prepare and Cook Food* in more detail. The plumbing and electrical components of storing, preparing and cooking food (such as sinks taps, hot water and stoves and ovens) all achieved between 90–100% compliance by the end of the project. Less improvement was made in areas where kitchen benches and cupboards required replacement (primarily a financial restriction) and in refrigeration (which is considered a tenant responsibility in social housing in NSW).

**Table A2:** Percentage of houses where Critical Healthy Living Priority: *Improving Nutrition: Ability to Store, Prepare and Cook Food* was fully met for all NSW projects from 1998–2017.

	Survey ID 1		Survey ID 2	
Healthy Living Priority	Per cent	95% CI	Per cent	95% CI
Number of Kitchens	99.9	(99.8, 100)	100	(100, 100)
Bench Material	79.3	(78.0, 80.6)	88.4	(87.3, 89.5)
Splash back	67.4	(65.9, 68.9)	83.9	(82.7, 85.1)
Storage Area Above Bench	58.2	(56.6, 59.8)	71.8	(70.3, 73.3)
Kitchen Sink Spout	67.1	(65.6, 68.6)	95.7	(95.0, 96.4)
Sink Drainage	91.9	(91.0, 92.8)	99.0	(98.7, 99.3)
Stove Available	98.8	(98.4, 99.2)	99.6	(99.4, 99.8)
Hotplates and knobs	62.1	(60.5, 63.7)	93.6	(92.8, 94.4)
Oven working	73.6	(72.1, 75.1)	96.5	(95.9, 97.1)
Fridge/Freezer available	90.5	(89.5, 91.5)	93.5	(92.7, 94.3)
Freezer temp.	79.1	(77.7, 80.5)	83.7	(82.4, 85.0)
Fridge temp	62.1	(60.5, 63.7)	71.6	(70.1, 73.1)
Cold Water Available	95.0	(94.0, 96.0)	99.7	(99.4, 100)
Hot Water Available	88.4	(86.9, 89.9)	99.3	(98.9, 99.7)
Cold Water Tap	73.1	(71.1, 75.1)	99.0	(98.5, 99.5)
Hot water Tap	73.4	(71.4, 75.4)	98.7	(98.2, 99.2)

## Appendix C. Changes in House Condition between Survey Fix 1 and Survey Fix 2 for Each of the Critical Healthy Living Priorities by Category of House Condition (Good, Fair or Poor)

Figure A3 presents the results of 112 Housing for Health projects by critical Healthy Living Priorities (CHLP) for Survey-Fix 1 (SF1) and Survey-Fix 2 (SF2) between 1998 and 2017. The scores for each priority are measured by performance score categories rather than just the percentage of houses that were all OK (i.e., achieved a 100% score). The categories (and scores) are: Good (100%) shown in dark green; Fair (51–99%) shown in light green and Poor (<50%) shown in yellow. It demonstrates that whilst not all houses achieved a 100% score for the HLP criteria at SF2, there was still measurable improvement in each house after the HfH intervention. Notably, after SF2 there is a significant reduction (*P* < 0.01) in the percentage of houses only achieving 50% or less of the possible score (in yellow).

**Table A3:** Comparison of Survey-Fix 1 and Survey-Fix 2 data for each critical Healthy Living Priority by category of house condition (Good, Fair or Poor) based on house function from 1998–2017.

		Per Cent	
Critical Health Living Priority	Category of House Condition	Survey ID 1	Survey ID 2
Power, water, waste connected	Very Poor (0–50%)	1.7	0.1
	Poor to Fair (51–99%)	23.1	4.0
	Good (100%)	75.2	95.9
Electrical safety	Very Poor (0–50%)	21.0	0.5
	Poor to Fair (51–99%)	71.5	12.1
	Good (100%)	7.5	87.4
Gas safety	Very Poor (0–50%)	43.8	16.7
	Good (100%)	56.2	83.3
Structure and access	Very Poor (0–50%)	18.9	2.1
	Poor to Fair (51–99%)	58.6	21.0
	Good (100%)	22.5	76.9
Fire safety	Very Poor (0–50%)	33.6	7.5
	Poor to Fair (51–99%)	37.1	12.4
	Good (100%)	29.3	80.1
Shower working adequately	Very Poor (0–50%)	7.3	0.3
	Poor to Fair (51–99%)	53.8	7.1
	Good (100%)	38.8	92.6
Facilities to wash children	Very Poor (0–50%)	25.2	1.9
	Poor to Fair (51–99%)	8.2	3.3
	Good (100%)	66.6	94.8
Laundry services	Very Poor (0–50%)	8.2	0.9
	Poor to Fair (51–99%)	62.6	18.5
	Good (100%)	29.1	80.6
Flush toilet working	Very Poor (0–50%)	14.9	1.5
	Poor to Fair (51–99%)	21.9	3.9
	Good (100%)	63.2	94.7
All drains working	Very Poor (0–50%)	5.6	0.6
	Poor to Fair (51–99%)	73.0	27.2
	Good (100%)	21.3	72.2
Facilities to Store, Prepare & Cook food	Very Poor (0–50%)	8.1	1.0
	Poor to Fair (51–99%)	82.9	59.9
	Good (100%)	9.0	39.1

## Appendix D. Changes in House Function in Housing for Health (HfH) Projects over Time

Figure A4 shows changes in each of the critical Healthy Living Priorities (CHLP) between Survey –Fix 1 (SF1) and Survey-Fix 2 (SF2), separated into four five-year intervals to assess differences in house function and improvement over time for each CHLP.

The condition of houses pre-HfH intervention (represented by the SF1 data) improved slightly over time for some criteria. Trend analysis showed over the two decades that there were positive trends across five-year periods in: *Power, water, waste connected*; *Fire safety*; *Facilities to wash children (basin/bath/tub)*, and *Store, prepare & cook food*. There was a ‘minimal’ positive trend in: *Flush toilet working*, and *All drains working*, and no trend in: *Shower working adequately* and *Laundry services.*

At SF2 there were positive trends toward improvement post-intervention across five-year intervals in all CHLPs, particularly after 2002. This trend toward improvement in CHLP scores at SF2 after 2002 was true for *Power, Water and Waste Connected*; *Electrical Safety*; *Gas safety*; *Shower working* adequately, and *Facilities to wash children (basin/bath/tub)*. There was also a significant improvement in the percentage of houses meeting the *Fire Safety*; *Structure and Access*, and *Store, Prepare and Cook Food* criteria in the second decade of the program compared to the first.

The data for Figure A4 is shown below in Table A4.

**Table A4:** Critical Healthy Living Priorities at Survey-Fix1 and Survey–Fix 2 by 5-year intervals from 1998-2017.

		Survey ID 1		Survey ID 2	
Healthy Living Priority	5-Year Period	Per cent	95% CI	Per cent	95% CI
Power, water, waste connected	1998 to 2002	65.1	(62.3, 67.9)	90.3	(88.5, 92.1)
	2003 to 2007	77.9	(75.2, 80.6)	97.2	(96.1, 98.3)
	2008 to 2012	80.5	(77.6, 83.4)	99.9	(99.6, 100)
	2013 to 2017	81.5	(78.9, 84.1)	98.1	(97.1, 99.1)
Electrical safety	1998 to 2002	10.1	(8.3, 11.9)	73.2	(70.5, 75.9)
	2003 to 2007	5.1	(3.7, 6.5)	93.4	(91.8, 95)
	2008 to 2012	9.1	(7.0, 11.2)	98.9	(98.1, 99.7)
	2013 to 2017	5.2	(3.7, 6.7)	89.1	(86.9, 91.3)
Gas safety	1998 to 2002	64.3	(58.1, 70.5)	65.8	(59.1, 72.5)
	2003 to 2007	55	(47.3, 62.7)	91.2	(86.5, 95.9)
	2008 to 2012	36.5	(25.5, 47.5)	100	
	2013 to 2017	54.5	(46.0, 63.0)	97.8	(94.9, 100)
Structure and access	1998 to 2002	52.7	(49.8, 55.6)	69.5	(66.7, 72.3)
	2003 to 2007	14.0	(11.8, 16.2)	69.1	(66.0, 72.2)
	2008 to 2012	7.0	(5.2, 8.8)	89.8	(87.6, 92.0)
	2013 to 2017	4.6	(3.2, 6.0)	83.5	(80.9, 86.1)
Fire safety	1998 to 2002	19.8	(16.5, 23.1)	71.8	(68.0, 75.6)
	2003 to 2007	19.3	(16.7, 21.9)	66.7	(63.6, 69.8)
	2008 to 2012	29.1	(25.8, 32.4)	88.8	(86.5, 91.1)
	2013 to 2017	46.9	(43.5, 50.3)	93.0	(91.2, 94.8)
Shower working adequately	1998 to 2002	37.9	(35.1, 40.7)	83.6	(81.3, 85.9)
	2003 to 2007	38.8	(35.6, 42.0)	96.3	(95.0, 97.6)
	2008 to 2012	36.2	(32.7, 39.7)	98.9	(98.1, 99.7)
	2013 to 2017	42.4	(39.0, 45.8)	94.6	(93.0, 96.2)
Facilities to wash children	1998 to 2002	52.1	(49.1, 55.1)	86.2	(84.1, 88.3)
	2003 to 2007	66.1	(63.0, 69.2)	97.0	(95.9, 98.1)
	2008 to 2012	75.7	(72.6, 78.8)	99.7	(99.3, 100)
	2013 to 2017	78.0	(75.2, 80.8)	98.8	(98.0, 99.6)
Laundry services	1998 to 2002	28.1	(25.5, 30.7)	69.8	(67.0, 72.6)
	2003 to 2007	28.4	(25.5, 31.3)	82.1	(79.6, 84.6)
	2008 to 2012	32.7	(29.3, 36.1)	87.1	(84.6, 89.6)
	2013 to 2017	28.1	(25.0, 31.2)	87.1	(84.7, 89.5)
Flush toilet working	1998 to 2002	56.3	(53.4, 59.2)	87.9	(85.9, 89.9)
	2003 to 2007	65.7	(62.6, 68.8)	96.7	(95.5, 97.9)
	2008 to 2012	68.2	(64.8, 71.6)	99.3	(98.7, 99.9)
	2013 to 2017	65.2	(62.0, 68.4)	97.3	(96.2, 98.4)
All drains working	1998 to 2002	13.4	(11.4, 15.4)	55.2	(52.2, 58.2)
	2003 to 2007	26.9	(24.0, 29.8)	75.9	(73.1, 78.7)
	2008 to 2012	26.3	(23.1, 29.5)	81.2	(78.3, 84.1)
	2013 to 2017	21.7	(18.9, 24.5)	82.5	(79.8, 85.2)
Facilities to Store Prepare & Cook food	1998 to 2002	3.8	(2.7, 4.9)	16.5	(14.2, 18.8)
	2003 to 2007	10.9	(8.9, 12.9)	29.7	(26.7, 32.7)
	2008 to 2012	9.5	(7.4, 11.6)	57.4	(53.8, 61.0)
	2013 to 2017	13.5	(11.2, 15.8)	62.9	(59.5, 66.3)

## Appendix E. Data Tables for Figure 2, Figure 3 and Figure 5

Table A5 provides the data for Figure 3: Percentage of houses with Critical Healthy Living Priorities fully met at Survey-Fix1 and Survey–Fix 2 for all NSW projects (*n* = 112) from 1998–2017 in the main text.

**Table A5:** Percentage of houses with Critical Healthy Living Priorities fully met at Survey-Fix1 and Survey–Fix 2 for all NSW projects (*n* = 112) from 1998–2017.

	Survey ID 1		Survey ID 2	
Healthy Living Priority	Per cent	95% C.I.	Per cent	95% C.I.
Power, water, waste connected	75.2	(73.8, 76.6)	95.9	(95.2, 96.6)
Electrical safety	7.5	(6.6, 8.4)	87.4	(86.3, 88.5)
Gas safety	56.2	(52.2, 60.2)	83.3	(79.9, 86.7)
Structure and access	22.5	(21.1, 23.9)	76.9	(75.5, 78.3)
Fire safety	29.3	(27.7, 30.9)	80.1	(78.6, 81.6)
Shower working adequately	38.8	(37.2, 40.4)	92.6	(91.7, 93.5)
Facilities to wash children	66.6	(65.1, 68.1)	94.8	(94.0, 95.6)
Laundry services	29.1	(27.6, 30.6)	80.6	(79.3, 81.9)
Flush toilet working	63.2	(61.6, 64.8)	94.7	(93.9, 95.5)
All drains working	21.3	(20.0, 22.6)	72.2	(70.7, 73.7)
Facilities to Store Prepare & Cook food	9.0	(8.1, 9.9)	39.1	(37.5, 40.7)

Table A6 provides the data for Figure 2: Average Percentage of houses with Critical Healthy Living Priorities fully met at Survey-Fix1 and Survey–Fix 2 for all NSW projects 1998–2017 by five-year intervals.

**Table A6:** Average Percentage of houses with Critical Healthy Living Priorities fully met at Survey-Fix1 and Survey–Fix 2 for all NSW projects 1998-2017 by 5-year intervals.

	Survey ID 1		Survey ID 2	
5-Year Period	Per cent	95% C.I.	Per cent	95% C.I.
1998 to 2002	35.2	(34.3, 36.1)	70.2	(69.3, 71.1)
2003 to 2007	35.7	(34.7, 36.7)	80.6	(79.8, 81.4)
2008 to 2012	37.4	(36.3, 38.5)	90.2	(89.5, 90.9)
2013 to 2017	39.0	(38.0, 40.0)	88.8	(88.1, 89.5)

Table A7 provides the data for Figure 5: Percentage of houses with Critical Healthy Living Priorities fully met for NSW projects with repeat visits at First Project SF1 (*n* = 802) and SF2 (*n* = 722); and at Second Project SF1 (*n* = 788) and SF2 (*n* = 734) from 1998–2017 in the main text.

**Table A7:** Percentage of houses with Critical Healthy Living Priorities fully met for NSW projects with repeat visits at First Project SF1 (*n* = 802) and SF2 (*n* = 722); and at Second Project SF1 (*n* = 788) and SF2 (*n* = 734) from 1998–2017.

		Survey ID 1		Survey ID 2	
Healthy Living Priority	Visit	Per cent	95% C.I.	Per cent	95% C.I.
Power, water, waste connected	1st visit	64.8	(61.5, 68.1)	92.9	(91, 94.8)
	2nd visit	81.9	(79.2, 84.6)	98.2	(97.2, 99.2)
Electrical safety	1st visit	4.5	(3.1, 5.9)	84.1	(81.4, 86.8)
	2nd visit	7.4	(5.6, 9.2)	92.2	(90.3, 94.1)
Gas safety	1st visit	63.7	(55.9, 71.5)	85.0	(78.0, 92.0)
	2nd visit	42.9	(32.3, 53.5)	98.4	(95.3, 100)
Structure and access	1st visit	33.7	(30.4, 37.0)	61.6	(58.1, 65.1)
	2nd visit	4.8	(3.3, 6.3)	83.0	(80.3, 85.7)
Fire safety	1st visit	10.0	(7.5, 12.5)	56.3	(52.1, 60.5)
	2nd visit	35.3	(32.0, 38.6)	87.7	(85.3, 90.1)
Shower working adequately	1st visit	34.6	(31.3, 37.9)	89.6	(87.4, 91.8)
	2nd visit	38.3	(34.9, 41.7)	95.9	(94.5, 97.3)
Facilities to wash children	1st visit	52.0	(48.5, 55.5)	92.8	(90.9, 94.7)
	2nd visit	75.9	(72.9, 78.9)	99.0	(98.3, 99.7)
Laundry services	1st visit	23.8	(20.9, 26.7)	74.0	(70.8, 77.2)
	2nd visit	31.1	(27.9, 34.3)	87.2	(84.8, 89.6)
Flush toilet working	1st visit	55.8	(52.4, 59.2)	92.2	(90.2, 94.2)
	2nd visit	63.8	(60.4, 67.2)	97.7	(96.6, 98.8)
All drains working	1st visit	15.2	(12.7, 17.7)	60.1	(56.5, 63.7)
	2nd visit	22.8	(19.9, 25.7)	82.3	(79.5, 85.1)
Facilities to Store Prepare & Cook food	1st visit	7.6	(5.8, 9.4)	21.1	(18.1, 24.1)
	2nd visit	10.3	(8.2, 12.4)	54.6	(51.0, 58.2)
